# The elusive concept of sexual motivation: can it be anchored in the nervous system?

**DOI:** 10.3389/fnins.2023.1285810

**Published:** 2023-11-17

**Authors:** Elisa Ventura-Aquino, Anders Ågmo

**Affiliations:** ^1^Escuela Nacional de Estudios Superiores, Unidad Juriquilla, UNAM, Juriquilla, Mexico; ^2^Department of Psychology, University of Tromsø, Tromsø, Norway

**Keywords:** sexual motivation, operational definition, sexual approach, erection, vaginal lubrication, brain

## Abstract

Sexual motivation is an abstract concept referring to the mechanisms determining the responsivity to sexually relevant stimuli. This responsivity determines the likelihood of producing a sexual response and the intensity of that response. Both responsivity to stimuli and the likelihood of making a response as well as the intensity of response are characteristics of an individual. Therefore, we need to assume that the concept of sexual motivation materializes in physiological mechanisms within the individual. The aim of the present communication is to analyze the requisites for the endeavor to materialize sexual motivation. The first requisite is to provide an operational definition, making the concept quantifiable. We show that parameters of copulatory behavior are inappropriate. We argue that the intensity of sexual approach behaviors provides the best estimate of sexual motivation in non-human animals, whereas the magnitude of genital responses is an exquisite indicator of human sexual motivation. Having assured how to quantify sexual motivation, we can then proceed to the search for physiological or neurobiological underpinnings. In fact, sexual motivation only manifests itself in animals exposed to appropriate amounts of gonadal hormones. In female rats, the estrogen receptor α in the ventrolateral part of the ventromedial nucleus of the hypothalamus is necessary for the expression of sexual approach behaviors. In male rats, androgen receptors within the medial preoptic area are crucial. Thus, in rats sexual motivation can be localized to specific brain structures, and even to specific cells within these structures. In humans, it is not even known if sexual motivation is materialized in the brain or in peripheral structures. Substantial efforts have been made to determine the relationship between the activity of neurotransmitters and the intensity of sexual motivation, particularly in rodents. The results of this effort have been meager. Likewise, efforts of finding drugs to stimulate sexual motivation, particularly in women complaining of low sexual desire, have produced dismal results. In sum, it appears that the abstract concept of sexual motivation can be reliably quantified, and the neurobiological bases can be described in non-human animals. In humans, objective quantification is feasible, but the neurobiological substrate remains enigmatic.

## Introduction

There is an old Chinese proverb saying: Without copulation, no population (Malmnäs, 1973). This statement is not universally true, but it is probably valid for all species with internal fertilization. In a more formal way, it can be stated that whenever an individual belonging to one of those species reproduces, there has been copulation. However, the reverse is not true, i.e., copulation does not necessarily lead to reproduction. In fact, the amount of copulatory behavior displayed by humans and other animals is far superior to that needed for reproduction. In humans, for example, it has been estimated that there are 1,100 copulations per birth, at least in Sweden (Lewin et al., 1998). Likewise, male and female rats display far more copulatory activity than needed for maximal reproduction (Chu and Ågmo, 2014, 2015; Chu et al., 2015). This is also the case in the primate species for which reliable data are available (Tutin, 1979; Nishida, 1997; Eberle and Kappeler, 2004; Watts, 2007; Stumpf and Boesch, 2010). Thus, there is a disparity between the amount of sexual behavior actually displayed by humans and other animals and that needed for maximal reproduction.

The disparity mentioned above can easily be explained if we stop to consider sex a reproductive behavior. In fact, since quite many years it has been generally accepted that humans and other animals copulate because that activity provides ephemeral pleasure (Boul et al., 2009, see Laan et al., 2021, for a review). Sex decoupled from reproduction can also be regarded as a leisure activity, not fundamentally different from other leisure activities (Berdychevsky and Carr, 2020). It can be maintained that for many humans, reproduction is an often undesired side effect of sexual pleasure. It is unknown if any animal other than the human ever has associated copulation with reproduction. It seems that nature, in its infinite wisdom, has assured that sex is so pleasurable that individuals of many species engage in far more sex than needed for maximal reproduction.

Because of the highly rewarding properties of sexual acts, sex is now regarded as a source of happiness and well-being (Anderson, 2013; Flynn et al., 2016; Mitchell et al., 2021). However, contrary to popular belief, the association between sexual frequency and happiness is curvilinear rather than linear. Wellbeing and frequency of sex are associated up to a frequency of once per week. Higher frequencies are not associated with greater well-being, at least not for people in relationships (Muise et al., 2015). Nevertheless, the absence of satisfactory sexual function reduces the quality of life (Naeinian et al., 2011; Bram Khemiri et al., 2020). This fact has stimulated the search for efficient treatments of sexual dysfunction (Frühauf et al., 2013; Nappi et al., 2022; Langarizadeh et al., 2023). Most unfortunately, with the exception of erectile deficiencies, this search has met with little success. There are not any established and efficient pharmacological treatment for the paraphilias (sexual motivation activated by unusual or socially unacceptable stimuli or bizarre sexual behaviors) nor for disabling lack of sexual interest. Likewise, there are no recognized treatment for hypersexuality. The therapeutic value of the drugs approved for the treatment of hypoactive sexual interest in the Unites States are questionable, at the best, with effects usually within the range of placebo treatment (Jaspers et al., 2016; Saadat et al., 2017; Anderson and Moffatt, 2018; Weinberger et al., 2019; Spielmans, 2022). They have all been commercial failures, something perhaps related to the dubious clinical effect.

One factor contributing to this sad state of affairs is that our knowledge of the mechanisms of sexual motivation, the fundamental process underlying all expressions of sexuality or the lack thereof, is faulty. We argue that the cause of the deficient understanding of the mechanisms of sexual motivation is twofold: On one side, the conceptual analysis of sexual motivation has been lacking in precision. It is even common to present extensive analyses of the concept of sexual motivation without offering a single proposal for an operational definition or propose such a definition in the form of responses to a questionnaire of some kind. On the other side, the neurobiological studies offer data as to the effects of some manipulation of nervous activity on a specific behavior pattern, for example the latency to ejaculation or the intensity of lordosis, without making any clear connection between that behavior pattern and the motivational mechanisms supposedly underlying it.

In this review we will try to connect an established conceptual model of sexual motivation with events in the brain. To that end, we will first operationalize rodent and human sexual motivation and then propose an abstraction of the central nervous mechanisms involved. This abstraction will then be anchored in the physical reality by showing that its manifestation can be altered by small molecules (gonadal hormones). We will also present arguments for proposing that these small molecules act in the central nervous system when determining sexual motivation, at least in rodents. Other small or large molecules (e.g., transmitters) will also modify the manifestations of the concept of sexual motivation. We will suggest that all these molecules will alter the activity of neurons, in the end the frequency of action potentials. The localization of some of the neurons involved in the materialization of the concept of sexual motivation is known in rodents, and we will venture into proposals of specific structures in the rodent brain as home for this abstract concept. We will end this review by asking whether detailed neurobiological knowledge of the mechanisms incarnating the concept of sexual motivation may be of any help for solving the problems posed by human sexual dysfunction. Whether the understanding of perfectly normal, unproblematic sexual behavior can be improved through the application of neurobiological data and neural models is another issue that will be approached.

## What is sexual motivation?

In vernacular language, sexual motivation can be defined as the urge to establish sexual contact with other individuals or to engage in solitary sexual activities, such as masturbation or watching pornographic movies. The intensity of the sexual urge can, in principle, vary from 0, i.e., absolutely no interest in sex of any kind, to some unknown maximum. In principle, the maximum should be reached when the individual is constantly engaged in seeking sexual contact or autoerotic activities, only interrupted by physiological needs such as micturition, defecation, drinking, eating, and sleeping. So far, there is no trustworthy report of an individual satisfying the criteria for having that level of motivation. It is also questionable whether there is any healthy human or other animal totally without sexual motivation. Regardless of the lack of experimental data concerning the upper and lower limits of sexual motivation, it can be postulated that it varies greatly both between and within individuals. The causes of the inter-and intraindividual variations in motivation are poorly known.

Motivation is an abstract concept, or intervening variable, used to explain the variable intensity of response to a constant stimulus. If we observe that a rat voraciously eats a large amount of the boring standard pellets when given the opportunity to do so, we assume that it is highly motivated to eat. When we communicate that observation to a lab colleague, we would probably use another expression, namely the rat is very hungry. The degree or intensity of hunger, motivation to eat, is not directly observable. Many potential physiological indicators of hunger have been proposed over the years, but none of them has been generally accepted. The gold standard for estimating the intensity of the motivation to eat has been, and is still, behavioral: The maximal effort accepted for obtaining food. This effort is usually estimated by employing a progressive ratio schedule with food reinforcement (e.g., Maske et al., 2018). Hunger, motivation to eat, is not in itself observable, but must be inferred from behavior. The concept of sexual motivation is similar to that of hunger, in the way that we need to use its behavioral manifestations to estimate its intensity. The nature of these manifestations as well as their quantification will now be examined in some detail.

The relationship between the abstract concept of motivation and the ensuing intensity of behavior becomes of paramount importance as soon as we want to study the factors, physiological or behavioral, underlying the manifestations of motivated behavior. The first step needed for making an abstract concept amenable for scientific study is to provide an operational definition. We maintain that sexual motivation *determines the probability of displaying sexual behavior when a mate is available or the intensity of that behavior when displayed. It can also refer to the intensity of approach to a potential sexual partner and to the magnitude of the genital response to sexually relevant stimuli* (Le Moëne and Ågmo, 2019, p. 2). The probability to display a behavior in a certain context is possible to determine experimentally, and so is the intensity of behavior. Likewise, the intensity of approach can be estimated with a high degree of precision, at least in non-human animals. Instead of evaluating the intensity of sexual approach in humans, we can quantify certain genital responses providing direct measures of the level of motivation.

The operational definition provided here refers to quantifiable variables. However, there are many ways to quantify each of these variables, both in human and non-human beings. Ideally, we should end up with reliable, valid, unambiguous, and unbiased measures of sexual motivation. Whether such measures exist or not is the subject of a later section. Before we can analyze that issue, we need to describe the sequence of events constituting a sexual encounter.

## A sexual encounter and an intervening variable

Behavior is always displayed in a context. Any context, even the simplest, contains a considerable number of stimuli. Most of these stimuli are neutral, meaning that they contain no useful information about future events in the context. Other stimuli may predict the availability of a reward. Such stimuli are called positive incentives. If the subject being in the context happens to be an intact male rat, the odor or the sight of a sexually receptive female rat predicts a potential sexual reward. Positive incentives activate approach behavior, and if approach to a sexual incentive is successful, copulatory activities may follow. A male rat, for example, will mount the female while performing antero-posterior pelvic thrusts. This may, or may not, bring the erect penis in contact with the vaginal orifice. In case contact is achieved, the male displays a deep forward thrust leading to vaginal penetration. After a few msecs he will dismount. After a short interval, he will resume mounting. After a couple of vaginal penetrations, usually between six and 10, he ejaculates. This is followed by several minutes of inactivity before resuming copulation.

The approaching male causes the female rat to show a paracopulatory behavior, a short run ended with a sudden stop and a presentation posture. The male finds this behavior very exciting and mounts the female. In fact, 98% of male mounts are preceded by a paracopulatory behavior (Bergheim et al., 2015). The tactile stimulation to the rump and flanks of the female provided by the mounting male causes her to display lordosis, a concave arching of the back, extension of the hind legs and movement of the tail to one side, exposing the vaginal opening (Pfaff et al., 1973). The mounting male may then penetrate the vagina. Females respond with lordosis to every male mount during the entire period of estrus (Chu and Ågmo, 2014).

The highly ordered sequence of acts constituting a rodent sexual encounter is displayed only when both participants are motivated to do so. In the absence of motivation, stimuli emitted by the female will have no incentive value, and the male will not approach her. In the absence of approach, there can be no sexual interaction. Similarly, if the female is not motivated, she will neither approach the male nor incite him to mount by displaying a paracopulatory behavior. She will not respond with lordosis to any mount attempts by an inconsiderate male. To explain the variations in responsiveness to the sexual incentive, it is extremely helpful to include a non-behavioral, intervening variable named motivation.

**Figure 1:**
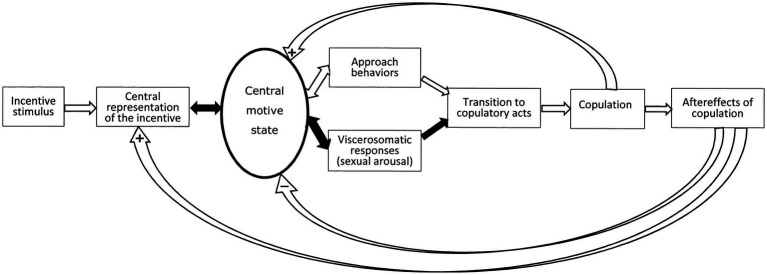
Schematic representation of the sequence of events during a sexual encounter according to the sexual incentive motivation model. For a detailed analysis of this model, see Ågmo and Laan (2023). The figure is reprinted from that paper under license CC BY.

The sequence of events and the intervening variable are illustrated in Figure 1. Here, the term motivation has been replaced by the notion of central motive state. At difference to motivation, the latter concept contains a hint as to possible localization, namely the central nervous system. When introducing the concept, Morgan (1942) defined it as a determinant of “general activity, specific behavior, and the readiness to perceive and react to stimulus situations in particular ways” (Morgan, 1942, p. 461). It can be maintained that the notion of central motive state is a materialization of the abstract concept of motivation. In addition to localize motivation to the central nervous system, Morgan’s notion further states that an essential feature of the central motive state is to determine the responsiveness to (readiness to perceive and react to) “stimulus situations in particular ways.” We consider that the determination of responsiveness to incentives, either positive or negative, is the basic function of motivational systems, hence of the central motive state.

If we consider that the essence of motivation is to determine responsiveness to incentives, we need to make explicit what responsiveness means. It might have a sensory component in the way that a high level of motivation facilitates the processing of relevant incentive stimuli, whereas a low level of motivation could be associated with inefficient processing. Responsiveness may also have a motor component, so that motor circuits are facilitated by high levels of motivation, or relevant visceral systems increase their level of activity when motivation is high. Finally, responsiveness may allude to the impact of relevant incentives on nervous activity in the somatic and visceral response systems, i.e., the transformation of sensory input into coordinated motor output is made more efficient. The central motive state can, consequently, be regarded as a kind of trinity, consisting of activity in sensory neurons leading to organized activation of central motor and neuroendocrine effector systems, canalizing their output to skeletal muscles through the somatic nervous system, to smooth muscles and some endocrine glands through the autonomic nervous system, and to other endocrine glands through the hypothalamic—adenohypophysial portal system as well as the neuroendocrine projections from the hypothalamus to the neurohypophysis. In this way, the concept of central motive state can be reduced to action potentials in neurons at specific brain sites. These action potentials are the result of many intraneuronal events, including activation of receptors for hormones and neurotransmitters. If the sexual encounter were successful, its positive properties will increase the likelihood of its repetition by anticipating reward and increasing the incentive value in subsequent encounters.

In this review we will try to describe where the action potentials forming the central motive state can be localized, and how they are controlled by hormones and transmitters. We will show that specific central nervous structures are crucial parts in this system, but a distributed network involving large portions of the brain and spinal cord, and even the peripheral nervous system, are needed for the full expression of sexual behavior.

## Quantification of sexual motivation

### Rodents

The statement “science has but one language, that of quantity, and but one argument, that of experiment” has been attributed to the British physiologist E. H. Starling (Berde, 1968, p. v). We completely agree with Starling. Therefore, we maintain that any scientific discussion of sexual motivation must be based on quantitative estimations of its intensity obtained in appropriately controlled experiments. Before addressing the issue of localization of the sexual central motive state and the neurobiological mechanisms responsible for the level of activity of that state, we need to analyze how to quantify the many manifestations of sexual motivation. Neither the concept of sexual motivation nor that of central motive state are directly observable, as mentioned several times already. As a result, they need to be inferred from observable behavior. The first problem, then, is to find a quantifiable behavior that reasonably can be expected to depend exclusively on the intensity of sexual motivation. This is a formidable task. We will illustrate this by mentioning the difficulties encountered when we want to obtain an uncontaminated, behavioral measure of sexual motivation in a rat.

Already in the 1970’s it was proposed that parameters describing copulatory behavior were inadequate for estimating the intensity of sexual motivation in rats. The argument was that these parameters, for example the latency to ejaculation or the intromission ratio, depend on additional factors, unrelated to sexual motivation *per se*. Among those are the activity of the striated penile muscles required for penile insertion, and the autonomous nervous system reflexes triggering seminal emission and ejaculation (Sachs, 1982; Clement and Giuliano, 2016; Soni et al., 2022). The constant oscillations between approach and withdrawal typical of rodent copulatory behavior further complicate the attribution of motivational significance to copulatory parameters (Le Moëne and Ågmo, 2019).

In addition to parameters of copulatory behavior as indices of sexual motivation, male rat erections stimulated by olfactory cues from females, the so-called non-contact erections (Sachs et al., 1994), as well as reflexive erections and spontaneous ejaculation sometimes have been suggested to be indicators of the level of motivation (e.g., Bancroft, 2005; Bialy et al., 2019). However, experimental data contradict such a proposal. Lesions of the preoptic area, eliminating sexual approach behavior as well as copulation [(Hurtazo et al., 2008) and references therein], have only marginal effect on non-contact erection (Liu et al., 1997). Likewise, penile reflexes as well as the frequency of spontaneous ejaculations are unaffected by preoptic lesions (Ågmo et al., 1977; Stefanick and Davidson, 1987). These and other observations strongly suggest that *ex copula* penile erection in rats is unrelated to the level of sexual motivation. This may be peculiar for rats, mice and some other rodent species, because erection in these species is dependent on contraction of the striated penile muscles (Giuliano et al., 1994; Bernabé et al., 1999; Soukhoval-O’Hare et al., 2007), *musculus ischiocavernosus* and *musculus bulbospongiosus*, whereas primate erection is a vascular response (Andersson and Wagner, 1995). This response is initiated when exposed to sexual incentives and lasts usually until ejaculation has been achieved. In rodents, erection is momentaneous, starting at the beginning of a mount and ending about 400 msec later when dismounting. At ejaculation, erection may last up to 2 s (Moralí et al., 2003). Rat and human erection are, then, abysmally different. While genital tumescence is a poor indicator of sexual motivation in rodents, it may be an exquisite indicator in primates, as will be discussed in a following section.

Instead of copulatory behaviors or penile responses, the level of sexual motivation should be inferred from the intensity of the urge to seek sexual contact (Meyerson and Lindström, 1973). This urge may be expressed as approach to a sexually active conspecific of the opposite sex. Meyerson and Lindström proposed three procedures for quantifying sexual approach (Malmnäs, 1973; Meyerson and Lindström, 1973; Meyerson et al., 1973; Hetta and Meyerson, 1978; Meyerson et al., 1979; Eliasson and Meyerson, 1981):

1. A straight runway ending in a compartment facing two doors surrounded by either horizontal or vertical black and white stripes. One door led to a cage containing a sexual incentive enclosed behind a wire mesh at one end. The other door led to a social incentive behind a wire mesh. The variables that could be recorded here were the time needed to traverse the runway and the time spent in the “choice compartment” before entering one of the cages as well as the proportion of trials in which the sexual incentive was chosen (e.g., Meyerson et al., 1973).
2. A two-cage setup with an electrified grid separating the cages. The experimental subject was put in one cage and had to traverse an electrified grid before entering the second cage, in which a conspecific was found behind a wire mesh. This could be either a sexually receptive animal of the opposite sex, or a sexually inactive animal of the same or the opposite sex. The intensity of the electrical current was increased each time the subject traversed the grid. The intensity required to prevent the subject from crossing the grid was the measure of motivation.
3. A circular arena in which four conspecifics were enclosed in small, half circular wire mesh enclosures placed at regular intervals on the inside of the arena wall. The conspecifics were always an intact male, a castrated male, a sexually receptive female, or a non-receptive (ovariectomized) female. This procedure offered simultaneous, limited contact to sexual and social incentives of both sexes without any prior training. The time spent in the vicinity of the sexual incentive relative to that spent in the vicinity of the other incentives constitutes the measure of motivation. This procedure was designed for evaluating the once popular notion of “sexual orientation” in addition to motivation.

**Figure 2:**
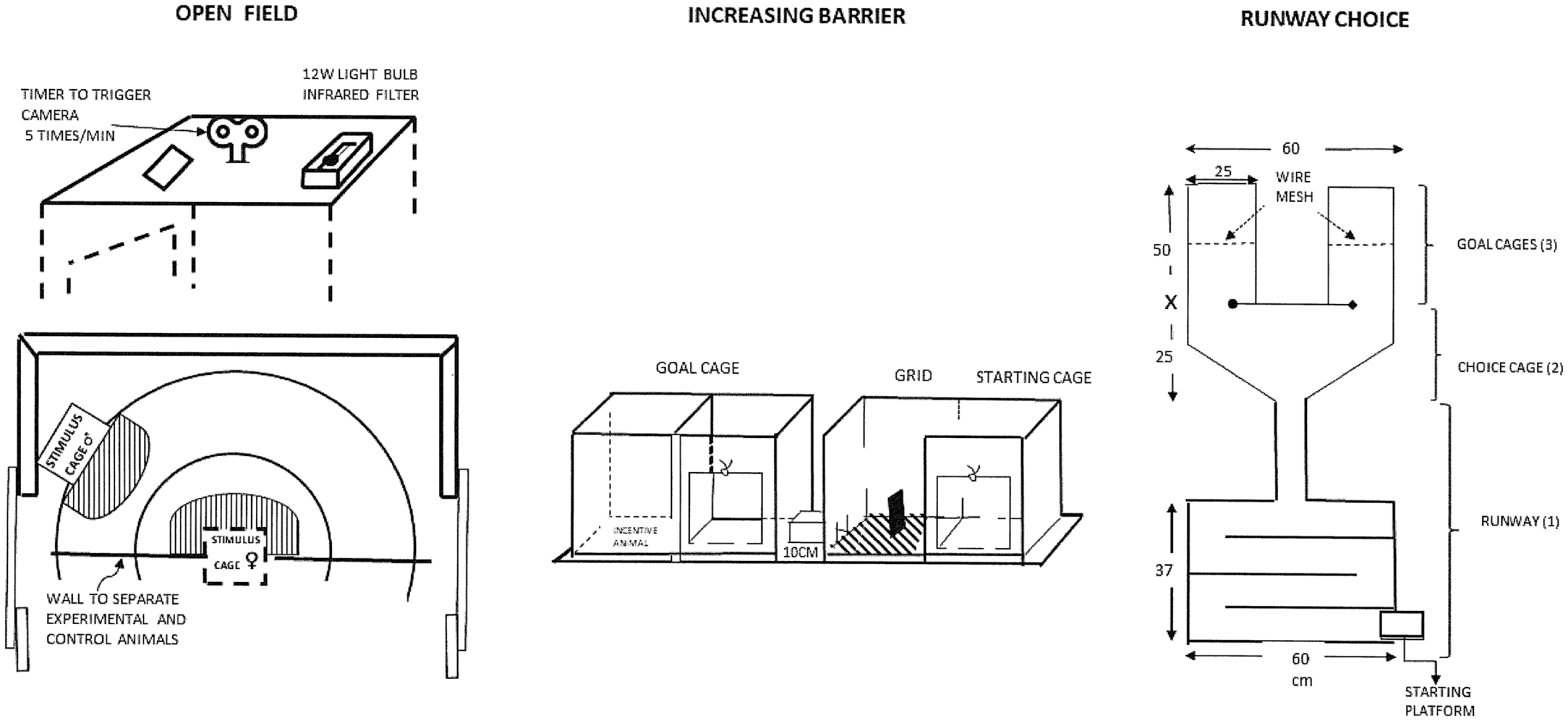
Drawings of the procedures used to measure sexual motivation in the pioneering studies of the Meyerson group in Uppsala. For a detailed description, see Meyerson and Lindström (1973). Reprinted from Meyerson et al. (1973) with permission from Elsevier.

The three procedures described above are illustrated in Figure 2. An extremely important feature of all these procedures is that copulatory activities never occurred because of the physical separation between the incentive and the experimental subject. Thus, sexual approach is never obscured by copulatory acts.

The extensive training or large number of trials needed for the first two procedures described above, and the influence of confounding variables, made them unsuitable for routine use. They rapidly entered the oblivion of history, whereas the third procedure was found to be both simple in use and providing reliable data. Above all, the data were easy to interpret even though there are multiple possible motives for approaching another rat. In addition to being a necessary prelude to sexual interaction (sex at a distance does not exist in rats), rats may approach others because they are seeking social contact, searching for heat from another body when environment is cold, or when seeking protection from fear-inducing, environmental events. By providing a non-sexual, social incentive it is possible to control for the three latter motives. They are equally, or perhaps even better, satisfied by a sexually inactive than by a sexually active rat.

**Figure 3:**
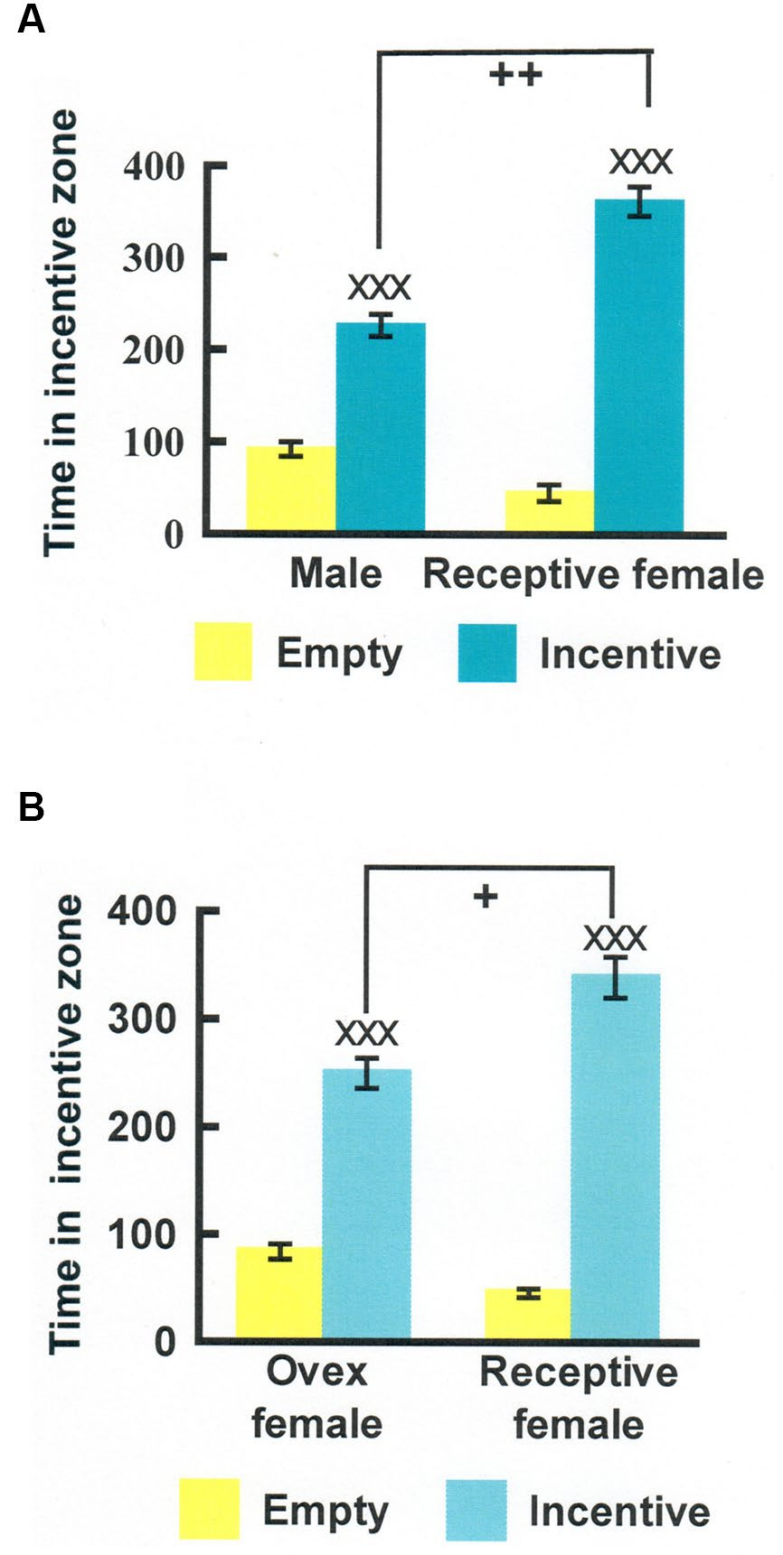
**(A)** Time (seconds) that male rats spent in the incentive zones in an experiment where either one incentive cage was empty and the other contained another male (Male) or one cage was empty and the other one contained a sexually receptive female (Receptive female). **(B)** Similar data from an experiment where the choices were either between an empty cage and an ovariectomized, nonreceptive female (Ovex female) or between an empty cage and a receptive female (Receptive female). ^XXX^Different from the empty cage, *p* < 0.001; ^++^Different from the time spent in the male incentive zone in the test with empty cage—another male, *p* < 0.01; ^+^Different from the time spent in the ovariectomized female incentive zone in the test with empty cage—ovariectomized female, *p* < 0.05. Modified from Ågmo et al. (2004), with permission from Elsevier.

A simplified version of the 3rd procedure mentioned above was developed by Josefa Vega-Matusczyk and Knut Larsson in Göteborg (Vega-Matuszczyk and Larsson, 1991, 1993). They employed two incentives only, one a potential sexual partner, the other a purely social partner. We have subsequently employed this simplified version in a considerable number of experiments. In an intent to verify that this procedure indeed can be used to quantify sexual motivation, several control experiments were performed. In one, intact male rats were provided a sexually receptive female and an empty cage as incentives. They spent a lot more time in the vicinity of the female than in the vicinity of an empty cage. This could be due either to search for social contact, for sexual contact or a combination of both. The intact males were also exposed to another male and an empty cage as incentives. They spent more time near the male incentive than near the empty cage. Provided that the experimental males were heterosexual, they must have approached another male because of social motivation. The experiment was performed at the typical ambient temperature in the animal room, making it unlikely that the experimental male was searching for heat from the other male. Likewise, no frightening event had occurred, making it unlikely that they searched for protection. When data from the two experimental conditions (receptive female vs. empty cage and another male vs. empty cage) were analyzed, it turned out that the experimental males spent significantly more time close to the female than to the male whereas there was no difference between the empty cages. Data from this experiment is shown in Figure 3. If we subtract the time spent near the other male from the time spent near the female, we get a measure of sexual motivation uncontaminated by social motivation.

Instead of running two experimental conditions, we could simply employ the incentives another male—sexually receptive female and immediately get the difference between approach to a social and a social + sexual incentive. We could even express that difference as a ratio, where 0.5 means the absence of sexual motivation whereas 1 is maximal sexual motivation (Ågmo et al., 2004).

**Figure 4:**
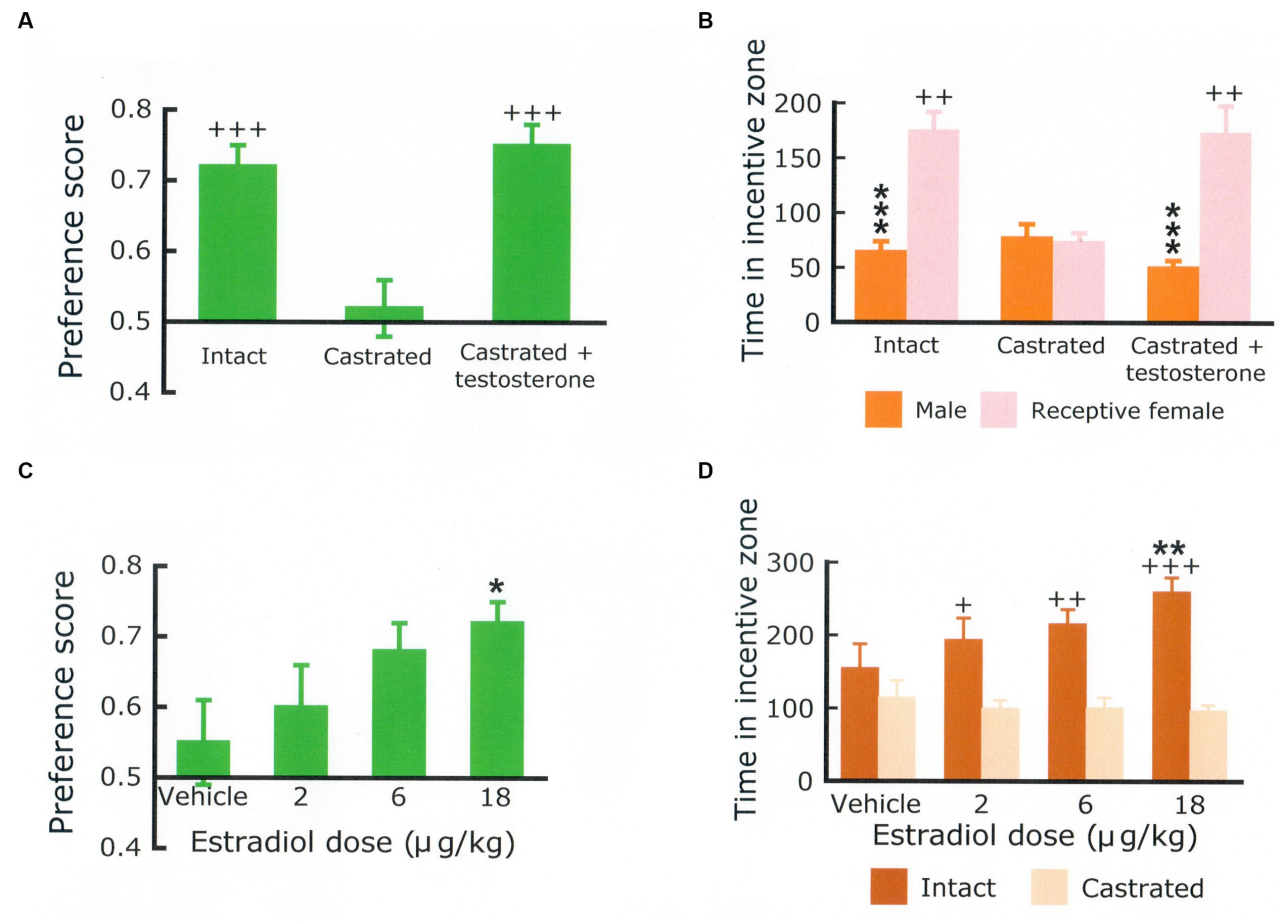
Preference score **(A)** and time (in seconds) spent in the vicinity of a social incentive (another male rat) and in the vicinity of a sexual incentive (sexually receptive female) **(B)** in a group of intact male rats (Intact), a group of males that had been castrated for at least 30 days (castrated) and a group of castrated males implanted with a testosterone-containing Silastic® capsule 20 days before the test. There were 12 males per group. Statistics: Main effect of treatment on the preference score, *F*(2,33) = 10.691, *p* < 0.001. Main effect of treatment on the time spent with the incentives, *F*(2,33) = 3.878, *p* = 0.031. Main effect of incentive, *F*(1,33) = 44.419, *p* < 0.001; interaction incentive × treatment, *F*(2,33) = 12.400, *p* < 0.001. ^***^Different from the female, *p* < 0.001, ^++^different from the castrated group, *p* < 0.01, ^+++^*p* < 0.001. The preference score **(C)** and the time spent in the vicinity of either a castrated or an intact male **(D)** obtained in ovariectomized females treated with varying doses of estradiol benzoate + progesterone. *N* = 16. Statistics: Main effect of estradiol dose on the preference score, *F*(3,45) = 3.511, *p* = 0.023. Main effect of dose on time spent with the incentives, *F*(3,45) = 2.800, *NS*; Main effect of incentive, *F*(1,15) = 12.390, *p* < 0.01; interaction dose x incentive, *F*(3,45) = 3.274, *p* < 0.05. Hormone treatment did not affect the time spent with the castrated male, whereas the time spent with the intact male was dose-dependently increased. ^*^Different from the vehicle, *p* < 0.05; ^**^p < 0.01; ^+^Different from castrated male, *p* < 0.05; ^++^*p* < 0.01, ^+++^*p* < 0.001. Data are mean ± S.E.M. The male data come from Attila et al. (2010) and the female data from Spiteri and Ågmo (2006). Procedural details are found in these papers.

**Figure 5:**
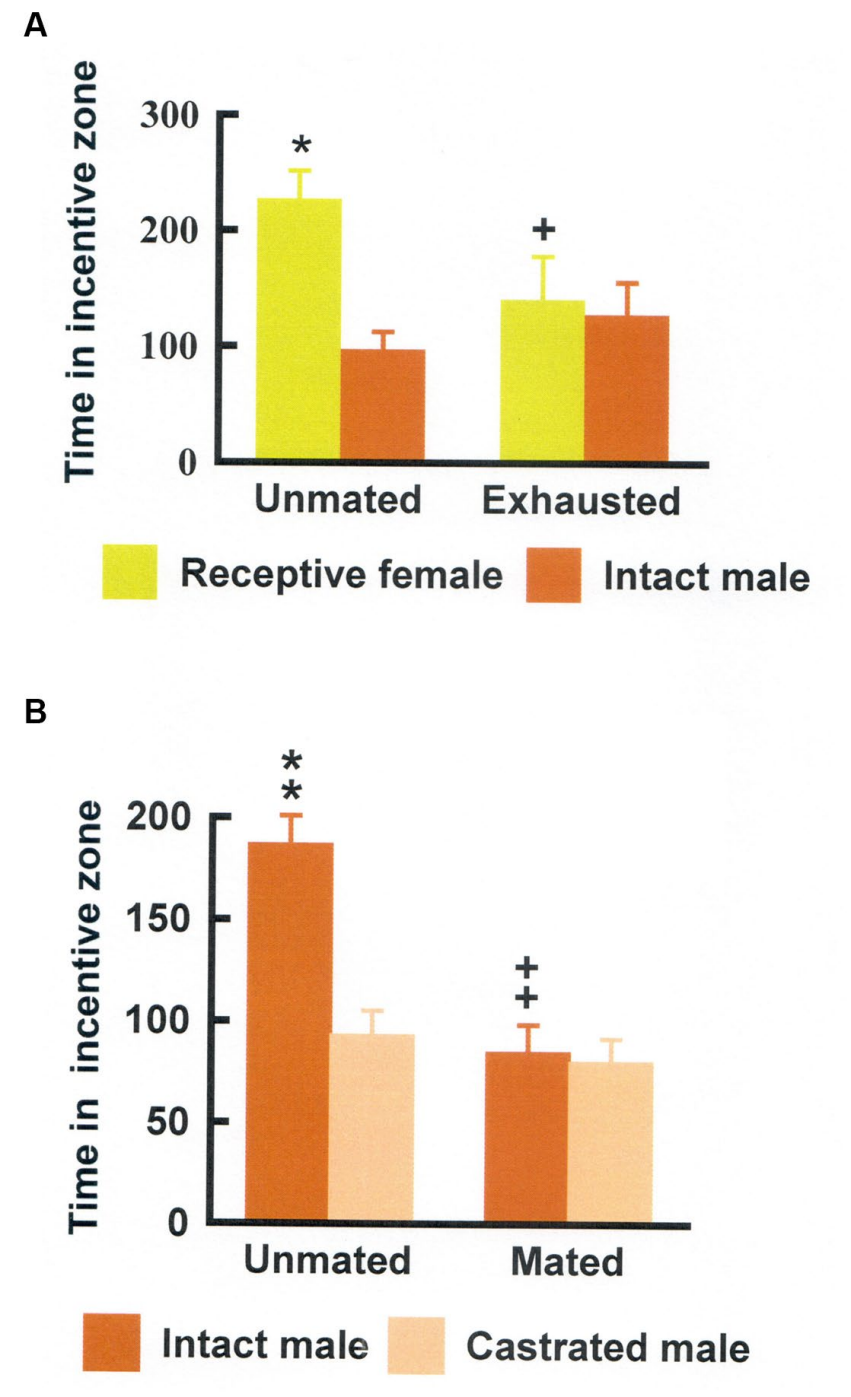
**(A)** Time (seconds) that male rats spent in the incentive zones either without preceding sexual activity (Unmated) or after copulating for 4 h (Exhausted). Data are mean ± S.E.M. ^*^Different from intact male, *p* < 0.05; ^+^Different from unmated, *p* < 0.05, ^++^*p* < 0.01, and ^+++^*p* < 0.001. **(B)** Time (seconds) that female rats spent in the incentive zones either without preceding sexual activity (Unmated) or after having received three ejaculations in a standard mating test (Mated). Data area mean ± S.E.M. ^**^Different from the castrated male, *p* < 0.01; ^++^Different from the time spent in the intact male incentive zone when unmated, *p* < 0.01. Modified from Ågmo et al. (2004), with permission from Elsevier.

This procedure has been further validated in several ways. First, it was reported that approach to a sexual incentive was no larger than approach to a social incentive in male and female rats deprived of gonadal hormones (Ågmo, 2003b; Spiteri and Ågmo, 2006). Superior approach to the sexual incentive could be restored by treatment with testosterone and estradiol + progesterone, respectively. The effects of gonadal hormones in males and females are shown in Figure 4. Furthermore, approach to the sexual incentive equals that to the social incentive in male and females immediately after having displayed intense sexual activity (having had free access to a sexually receptive female for 4 h in males, received three ejaculations in females; Ågmo et al., 2004). The results of this experiment are illustrated in Figure 5. Neither the presence or absence of gonadal hormones nor preceding sexual activity altered approach to the social incentive, showing that these manipulations specifically reduce sexual motivation.

It must be added that the procedure described above is quite insensitive to motor disturbances. There are drugs that strongly enhance motor activity without modifying sexual motivation, and there are drugs causing profound reductions of activity while leaving sexual motivation unaffected (Ågmo, 2003a; Ellingsen and Ågmo, 2004). Even doses that impair gross motor coordination, as evaluated on a rotarod, leaves approach behavior unaffected, as long as the experimental subject is able to move at all (Ågmo and Fernández, 1989; Ågmo, 2003a). There are also drugs that strongly reduce ambulatory activity and simultaneously enhance sexual motivation (Viitamaa et al., 2006; Chu and Ågmo, 2016). The independence of effects on locomotor activity and on sexual motivation is extremely important for avoiding false conclusions. Many drugs and other manipulations thought to modify sexual motivation also modify motor functions, making any direct effect on motivation questionable (see Paredes and Ågmo, 2004, for an extensive discussion).

Different versions of this basic procedure have been described. Some prefer to measure the frequency of nose pokes to different incentives rather than to measure the simple time spent near the incentive (Cummings and Becker, 2012; Shimomi and Kondo, 2020; Hernandez-Gonzalez et al., 2022). The advantage of this is unknown, but the disadvantage is immediately obvious: Nose pokes are very sensitive to alterations in exploratory activity, and sedative effects of a treatment would be confounded with reduced motivation whereas stimulatory effects on locomotor activity would be confounded with enhanced sexual motivation. This kind of faulty procedures underlies perhaps the widespread misunderstanding that dopaminergic stimulants enhance sexual motivation [reviewed in Paredes and Ågmo (2004)]. Others use the time sniffing the wire mesh separating the incentive from the experimental animal as measure of motivation. This entails the same problems with motor activity as nose pokes. We will not provide an exhaustive review of all the methods that have been used in the belief that they may provide useful data concerning sexual motivation. This has been done elsewhere (Ventura-Aquino and Paredes, 2017).

To summarize this discussion, we propose that quantifications of approach to a sexual incentive corrected for approach to a social incentive can be used as an approximate measure of the intensity of sexual motivation. The observation of approach in the absence of copulatory interaction is required for an unbiased estimate of sexual motivation. Finally, the procedure employed should be insensitive to modest alterations in locomotor activity and motor coordination.

### Humans

In principle, the intensity of sexual approach behaviors should be possible to evaluate in humans. However, this possibility has not been exploited. Instead, studies of sexual motivation in humans have employed questionnaires of different kinds. One frequently used questionnaire (Spector et al., 1996) includes items like “when you have sexual thoughts how strong is your desire to engage in sexual activity with a partner” or “during the last month, how often would you have liked to engage in sexual activity with a partner (for example, touching each other’s genitals, giving or receiving oral stimulation, intercourse, etc.).” A problem with the use of questionnaires or other kinds of self-reports for inferring the intensity of sexual motivation is that the relationship between questionnaire responses and self-reports on one side and actual behavior on the other is uncertain. In fact, questionnaires are notoriously unreliable when it comes to surveys of sexual behaviors (Levin and Wagner, 1985), and responses seem to be heavily influenced by social desirability and other irrelevant factors (e.g., Beaussart and Kaufman, 2013; Krumpal, 2013; Milani et al., 2019; Kellie et al., 2020; Suschinsky et al., 2020). Some attempts have been made to reduce the influence of social desirability by using the “bogus pipeline” technique, in which participants are hooked up to a nonfunctioning polygraph making them believe that dishonest responses will be detected (Alexander and Fisher, 2003). The employment of this technique to reduce response bias is rare, and its reliability is still unknown. Thus, we suggest that questionnaires for quantifying the intensity of sexual motivation do not provide usable data.

In the figure illustrating the sequence of events during a sexual encounter (Figure 1) there is an arrow pointing from the central motive state to visceral responses. The most prominent of these are penile and clitoral engorgement. The many other responses, such as enhanced heart and respiratory rates, increased blood pressure, pupil dilation and so on are manifestations of the increase in general arousal caused by heightened activity in the sexual central motive state (Ågmo and Laan, 2022). These responses are common for many different motivational states and cannot be used for estimating the intensity of sexual motivation. However, the genital responses are specific consequences of enhanced activity in the sexual central motive state. Enhanced general arousal does not produce any genital response *per se* in human males (Wolchik et al., 1980) or females (Laan et al., 1995a), although it may enhance the response to sexual incentives (Meston and Gorzalka, 1995; Meston, 2000). In addition to being specific for sexually relevant stimuli, the genital responses are outside of voluntary control. The connection between the sexual central motive state and genital responses is automatic (unconscious), consequently non-volitional. As pointed out already by St. Augustine in the 4th century, a man can neither suppress an inconvenient erection with his will, nor use the will to cause an erection when needed. Likewise, the experience of orgasm can neither be created nor inhibited by acts of will. The automaticity of genital responses has been carefully studied (Laan and Everaerd, 1995; Laan et al., 1995b). We propose that the genital responses offer an unbiased estimate of the activity in the sexual central motive state. The facts that some erections such as, for example, morning erections, occur without sexual motivation and that men sometimes are unable to achieve an erection despite high levels of sexual motivation do not invalidate this proposal. Furthermore, it can be mentioned that the magnitude of these responses has been shown to depend on the intensity of the sexual incentive stimulus.

**Figure 6:**
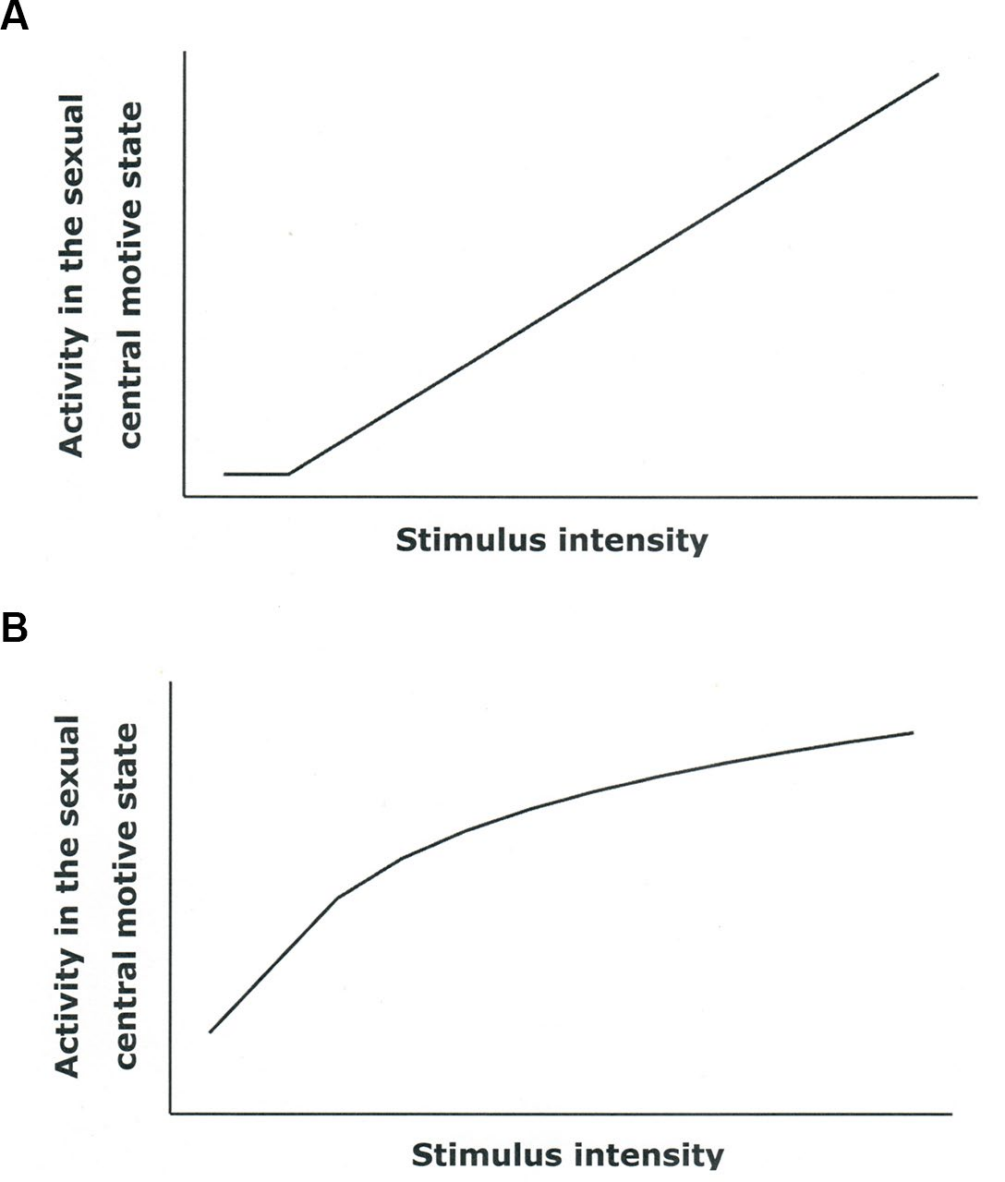
Hypothetical relationships between the activity of the sexual central motive state and the genital response. **(A)** There is a linear relationship. **(B)** The relationship is curvilinear.

In an unusually elegant study, Bouchard et al. (2019) exposed young women to video segments depicting sexual scenes of varying intensity (a heterosexual couple engaged in intimate caresses with clothes on or a naked couple engaged in penile-vaginal intercourse emitting vocalizations typical for the occasion). The control condition was a nature movie. Both video segments with sexual content enhanced vaginal blood flow, but the increase was larger during the video depicting intercourse. Similar data are available from young men (McConaghy, 1974; Gaither and Plaud, 1997). These observations strongly suggest that there is a direct relationship between the activity in the central motive state and the magnitude of the genital response as illustrated in Figure 6.

Objective measures of the genital response in men and women are readily available. In men, the genital response of erection is easily recorded. The most common procedure consists of fitting a strain gauge transducer to the base of the penis. The transducer is sensitive to changes in penile circumference, with increased circumference being an expression of erection and decreased circumference a sign of detumescence. The larger the change, the more intense is the erection or the detumescence, respectively. This procedure has been used since the 1960s (Bancroft et al., 1966; Barlow et al., 1970). It is known as penile plethysmography, although penile volume is not measured. It is also possible to actually quantify penile volume with an apparatus originally described by Freund et al. (1965). This kind of measurement is known as volumetric plethysmography, a rather absurd pleonasm. It is rarely used, even though measures of penile volume are more sensitive than measures of circumference (Freund et al., 1974; Kuban et al., 1999). Measures of penile skin temperature are also used in experimental settings (Tavares et al., 2018), but although reliable and reasonably sensitive they are still uncommon. Measures of penile rigidity are commonly employed in the clinic, but it is uncertain if such measurement provide any useful information above that obtained with measurements of circumference or volume (Rohrer et al., 2022). This short overview of methods to record the intensity of erection is not exhaustive, but it should be evident that there are several reliable methods easily available.

In women, a procedure called photoplethysmography has become dominant for measuring vaginal blood volume. The measure normally used, vaginal pulse amplitude, records the increase in vaginal blood volume from diastole to systole, i.e., during a heartbeat. This measure is far more sensitive than records of resting state vaginal blood volume (Geer et al., 1974; Laan and Everaerd, 1998). A photoplethysmograph can also be adapted to the clitoris (Mechelmans et al., 2020) providing a sensitive measure of the magnitude of the genital response. A different approach consists of directly recording vaginal blood flow with laser Doppler flowmetry (Bouchard and Pukall, 2023). This may be a promising procedure, but so far it has not been much used. There are several other procedures available (Tavares et al., 2018), but since this is not a methods paper we will not further mention them.

The preceding few paragraphs should have made clear that there are plenty of methods available for making quantitative recordings of genital responses in men and women. Since the magnitude of these responses is directly dependent on the intensity of sexual motivation, quantitative, objective measures are easily available. Furthermore, all the procedures mentioned in this section are non-invasive, making them possible to use in healthy human volunteers without ethical concerns. It could be maintained that the genital responses are godsent gifts to the scientists interested in objective measurements of human sexual motivation. It is difficult to understand why questionnaires still are used in studies of sexual motivation, despite their well-known limitations.

### Conclusion concerning the quantification of sexual motivation

According to Starling, and probably also to many others attached to the positivist or behaviorist tradition, quantification is a *sine qua non* for all scientific activity. Therefore, we have dedicated almost 3,000 precious words to this subject. In the case of non-human animals, we proposed that a specific kind of setup for evaluating approach behavior offers the best possible data. We immediately accept that this proposal may be offer of criticisms, but at the same time we have presented arguments that should be enough to suggest that our conclusion is not completely arbitrary. In the remaining part of this paper, we will limit our discussion of non-human sexual motivation to studies providing data on sexual approach behaviors, obtained in the absence of copulatory activity.

Concerning the human, we will only present data derived from genital responses. The conscious experiences documented in self-reports may perhaps be illustrative and useful for many purposes, but not so for studies of sexual motivation. If we really want to understand the mysteries of sexual motivation, it is preferable to record the activity of the sexual central motive state unaltered by conscious manipulation. Again, this choice can be criticized, but so can any other choice.

## Materialization of the concepts of motivation and central motive state

We have now described ways to operationalize the basic motivational concepts. One way to anchor them in physical reality could be to find out whether experimental alterations of some aspect of that reality manifest themselves in changes in motivation. For example, we could change the milieu interieur in an animal by removing the gonads. This would eventually lead to the disappearance from the body of the small molecules secreted by the sexual glands. If sexual motivation were altered, we would have shown that these small molecules, part of the physical reality, exert influence on the abstract concept. If altered activity of a transmitter would affect sexual motivation, we would have another example of a molecule, a rather fundamental piece of physical reality, exerting influence on the abstraction. If we also could modify sexual motivation through lesion of small or large parts of the brain, we would have localized the abstract concept to brain structures. We might even hope to alter motivation with the deletion of a gene, thereby offering additional support for the notion that motivation is determined by intracellular biochemical processes. Such deterministic relationships would show that the abstract concept can be perfectly anchored in physical reality, and consequently it is a respectable element in scientific reasoning.

In the following, we will provide several examples of the connection between the abstract concept of motivation and physical processes within the organism. These examples do not constitute an exhaustive review of all the evidence, but they are sufficient for our purpose.

### Gonadal hormones and sexual motivation

#### Rodents

It is perhaps a triviality to affirm that rodents show signs of sexual motivation only when their brain is exposed to adequate amounts of gonadal hormones. Castrated male rats do not approach a sexual incentive more than they approach a social incentive. Testosterone or dihydrotestosterone + estradiol restore sexual approach to a level indistinguishable from that of intact rats. Neither dihydrotestosterone alone, estradiol alone, nor testosterone combined with an aromatase inhibitor is effective (Attila et al., 2010). In male rats, then, the simultaneous activation of estrogen and androgen receptors are necessary for the manifestation of sexual approach behaviors. At present, there are no data as to the localization of hormone actions on sexual approach behavior in male rats. As is so often the case, this is in sharp contrast to the overwhelming amount of data concerning the localization of hormone actions on copulatory behavior (see Hull and Rodríguez-Manzo, 2017, for a review).

Male mice are, as always, a source of some confusion. Whereas whole-body knockout of the estrogen receptor α has been reported to eliminate preferential approach to the odor of receptive females in some studies (Rissman et al., 1997; Wersinger et al., 1997; Wersinger and Rissman, 2000), it has been found to be without effect on approach to female urine or vaginal secretions (Ogawa et al., 1996). Knockout of the androgen receptor within the nervous system of male mice leaves approach to sexual incentives either in the form of female odor (Raskin et al., 2009) or in the form of anogenital sniffing (Juntti et al., 2010) unaffected. Incidentally, it is questionable whether anogenital sniffing is an approach behavior, since this kind of sniffing is possible only after completed approach. Regardless of this, the role of gonadal hormones for sexual approach behaviors in male mice is unclear.

Here it is necessary to comment on the peculiar habit of some scientists to exclusively use olfactory stimuli as social and sexual incentives. Still worse, the preferred odor sources have been urine or bedding soiled by a potential incentive. It is most likely that soiled bedding contains substantial amounts of urine and feces. It has been argued that the use of excretion products as incentives is most unfortunate (Le Moëne and Ågmo, 2018). Instead, body odors emanating from a living animal should be used. In fact, such odors are more powerful incentives than excreta (Ågmo and Snoeren, 2017). In the natural habitat, body odors may be helpful for localizing a mate whereas odors from excreta only indicate that a potential mate was present sometime in the past. Nevertheless, in the absence of better data we often have to use approach to excreta as an approximation to approach to a mate.

In female rats, ovariectomy reduces the intensity of approach to a sexual incentive to the level of that shown for a social incentive. Sequential treatment with estradiol and progesterone dose-dependently increases approach to a sexual incentive without affecting approach to a social incentive (Spiteri and Ågmo, 2006). The simple experiments in male and female rats are sufficient for concluding that testicular and ovarian hormones are needed for the manifestation of sexual motivation in male and female rats, respectively. In females, it has also been shown that estradiol fails to enhance sexual motivation when the expression of the estrogen receptor α in the ventrolateral part of the ventromedial nucleus of the hypothalamus is blocked by administration of an iRNA directed against this receptor (Spiteri et al., 2010). In female mice, little is known about the endocrine regulation of sexual approach behaviors. One reason for this state of affairs are observations showing that female mice approach male olfactory stimuli with equal intensity throughout the estrus cycle (Scott and Pfaff, 1970; Hayashi and Kimura, 1978). This precludes any involvement of estrogens, hence estrogen receptors, in the immediate or short-term control of the intensity of sexual motivation. It even seems that female mice approach odor from males even several weeks after ovariectomy, suggesting that responsiveness to male stimuli is independent of estrogens (Moncho-Bogani et al., 2004). At the same time there are data showing that the firing frequency of neurons within the ventrolateral part of the ventromedial nucleus of the hypothalamus increased during interaction with a male, and that the increase was maximal when the experimental female was sexually receptive. Furthermore, no receptivity-associated increase was found when the female was interacting with another female (Nomoto and Lima, 2015). As far as can be judged from the incomplete procedure description, the female subjects were sexually naïve. Finally, it has been suggested that responses to olfactory stimuli from the opposite sex are hormone-dependent only in sexually inexperienced mice. Females with experience respond equally in the presence and absence of estrogens (McCarthy et al., 2018). One possible conclusion from all these data is that sexual motivation depends on estrogens in mice without sexual experience, whereas sexual motivation in experienced mice is independent of estrogens.

The fact that the functioning of the abstract concept of sexual motivation and the notion of a central motive state can be reliably altered by some steroid molecules acting at their cognate receptors in male and female rats suggests that both abstract concepts can be connected to a physical reality. Moreover, the additional fact that the actions of estradiol require an intact estrogen receptor α shows that the concepts can be anchored in neurons, since central estrogen receptors are expressed mainly in neurons (Pérez et al., 2003). Finally, since the effect of silencing this receptor is specific to the ventromedial nucleus of the hypothalamus, we can propose that the concept of sexual motivation is materialized in neurons within the ventromedial nucleus of the hypothalamus expressing the estrogen receptor α. This simple analysis of hormone effects on sexual motivation not only allows us to anchor the concepts of sexual motivation and central motive state in the brain but also to localize it to estrogen receptor α-expressing neurons in the ventromedial nucleus in rats. We do not, by any means, propose that sexual motivation in female rats is incarnated exclusively in the neurons and nucleus mentioned above. It is quite possible that future studies will reveal that the gonadal hormones are acting in distributed networks when controlling rat sexual motivation. Perhaps the concept of sexual motivation can be regarded as diffusely materialized in such networks. Regardless of that, there is no doubt that the discovery of the hormonal control of sexual motivation has contributed to anchoring the concept of motivation in the rat brain.

Data from male and female mice are too contradictory for proposing any convincing conclusion. The remarkable difference between the consistency of the rat data and the many inconsistencies in the mice data makes it clear that generalizations between species may be risky. It also illustrates that mice are not just small rats.

#### Humans

The role of gonadal hormones in the control of human sexual motivation has been and is still a matter of discussion. It has been pointed out that whereas motivational mechanisms can be stripped down to their essentials in laboratory animals, human sexual motivation is affected by cultural factors, possibly interacting with gonadal hormones in complex ways (Pfaff and Saad, 2020). Nevertheless, there are observation in humans relevant for sexual motivation as operationally defined in the present contribution. Data from men castrated because of prostate cancer and having very low serum concentrations of androgens show that the penile response to sexual incentives (a pornographic movie) is much reduced (Greenstein et al., 1995). This is also the case in men suffering from severe hypogonadism after treatment with leuprolide (Schober et al., 2005), a compound inhibiting gonadotropin release from the pituitary. Reduced penile response to sexual incentives in hypogonadal men clearly suggests that sexual motivation is hormone-dependent, perhaps even androgen-dependent. However, these studies give no clue to whether the putative motivational effects of androgens are localized to the central nervous system or not, and even less they offer information concerning the kind of cells involved. Despite considerable effort, we have been unable to find any data concerning the localization of hormone effects on sexual motivation in men. It is not known whether testosterone acts both via androgen and estrogen (after being aromatized) receptors or if actions at androgen receptors are enough. However, copulatory behavior depends exclusively on androgens acting at the androgen receptor in men and other primates (Phoenix, 1974; Bagatell et al., 1994; Gur et al., 2013; Sartorius et al., 2014), and we propose that this also is the case for sexual motivation in the human male.

The situation is still more precarious in women. Since females produce a lot more estrogens and less androgens than males, it appeared logical to search for a role of estrogens in the control of sexual motivation in women. However, data are mostly negative. If we stick to the objective measures described above, there is much evidence against a role for estrogens, and none for such a role. When the vaginal response to sexual incentives is evaluated during different phases of the menstrual cycle, no effect of cycle phase is found (Hoon et al., 1982; Morrell et al., 1984; Meuwissen and Over, 1992; Bossio et al., 2014). Furthermore, the vaginal response to pornographic movies is not altered by menopause (Laan and van Lunsen, 1997; Maravilla et al., 2003a,b; Suh et al., 2004) despite the strong reduction in circulating estrogens associated with that state. Since there are large variations in the serum concentration of estrogens between different phases of the cycle and between cycling and menopausal women, it must be concluded that estrogens do not determine sexual motivation in women, at least not when objective measures of motivation are used.

Ever since the publication of data showing that ovariectomized women who had also been subjected to adrenalectomy showed much reduced sexual function (Waxenberg et al., 1959) it has been suspected that androgens of adrenal origin are important for sexuality in women. Amenorrheic women have been reported to have lower serum testosterone concentration than cycling women (Tuiten et al., 1996), and their vaginal response to weak sexual incentives (fantasies and a movie segment showing sexual foreplay) was lower. When exposed to a strong sexual incentive (a movie segment showing intercourse) there was no group difference. This is a most interesting observation, since the activity of the sexual central motive state is a crucial determinant of the responsivity to sexual incentives. When activity is low, incentives need to be strong if they are to produce a response, whereas stimuli of high intensity produce a response anyway. Thus, the observation that females with low serum testosterone concentration responded less to a weak stimulus is exactly what should be expected if androgens were important for the responsivity of the sexual central motive state, hence for sexual motivation. Eight weeks of treatment of these women with testosterone undecanoate (40 mg daily) much enhanced serum testosterone concentration, reduced the level of sex hormone binding globulin, and increased the vaginal response to the sexual stimuli. This shows that testosterone indeed enhances the responsivity of the central motive state.

Acute testosterone treatment also enhances the vaginal response to sexual incentive stimuli, albeit with a delay of several hours between peak plasma concentration and peak stimulatory effect on vaginal vasocongestion (Tuiten et al., 2000, 2002; Heard-Davison et al., 2007; van Der Made et al., 2009). Such delays are to be expected considering that most actions of testosterone are genomic (Mhaouty-Kodja, 2018).

Since the importance of the vaginal response to sexual incentives has been underestimated as an unbiased indicator of the intensity of sexual motivation, data relevant for the effects of treatment with hormones or xenobiotics on this response are rare. Nevertheless, we consider that there are enough data for suggesting that testosterone is of some importance. Since the effects of estrogens seem to be modest, we further suggest that testosterone is acting at androgen receptors rather than on estrogen receptors (after aromatization). All the studies mentioned here employed systemic administration precluding any speculations concerning the site of action. Since androgen receptors have been described both in the vaginal epithelium, vestibule and labia minora (Palacios, 2020), a local action in vaginal tissue cannot be excluded.

#### Hormonal conclusion

Results of studies of the hormonal control of sexual motivation in rats provide compelling evidence for a role of the gonadal hormones. Furthermore, these hormones act on receptors on neurons in the brain. Whereas data from female rats allow for suggesting that the ventromedial nucleus of the hypothalamus is a crucial site of action, data from males do not allow for any speculations as to localization of hormone actions on sexual approach. In mice, data are too contradictory for proposing any role at all of gonadal hormones. In men and women, androgens may be needed for maintaining sexual motivation. Whether they act in the central or peripheral nervous system or elsewhere in the body is unknown.

The summary of hormone actions on sexual motivation presented above is incomplete in many aspects. Nevertheless, the purpose was not to provide a review of the endocrinology of sexual motivation, but to show that physical entities in the form of small molecules can determine the behavioral manifestations of the activity of the abstract concepts of sexual motivation and central motive state. Furthermore, we have tried to show that it sometimes is possible to use the experimental data to localize these concepts not only to a specific organ (the brain) but also to a specific cell type (neurons). We can even go a step further and propose that we can localize motivation and central motive state to neurons expressing the estrogen receptor α and/or the androgen receptor, at least in rats.

### Neurotransmitters and sexual motivation

#### Rodents

Most of the studies offering data on the effects of manipulations of transmitter systems on sexual behaviors have limited themselves to observations of copulation. In addition, results are often contradictory between studies. The old data have been summarized in several excellent reviews (Bitran and Hull, 1987; Dornan and Malsbury, 1989; Pfaus and Everitt, 1995; Hull et al., 1999; Argiolas and Melis, 2013), and we see no reason to repeat these summaries here. Because pharmacological studies of rodent sexual behavior are out of fashion, very little new data have appeared during the last decade. Here we will ignore all the data on copulatory behavior. Instead, we will focus on the small amount of data directly relevant for the issue of sexual motivation, i.e., data on sexual approach behaviors.

Amphetamine, in a dose of 1 mg/kg given to female rats, caused a robust increase in locomotor activity and a decrease in the time spent in the vicinity of a male in relation to the time spent close to a female (Guarraci and Clark, 2003). Even though the effect was of borderline significance (*p* = 0.05) it can be suggested that enhanced dopamine release reduce female sexual motivation. In a more extensive study, it was found that amphetamine did not affect female approach to a male, whereas this approach was reduced by the dopamine agonist apomorphine (Ellingsen and Ågmo, 2004). Dopamine antagonists, even at doses strongly reducing locomotor activity, were ineffective. Thus, available data show that increased dopaminergic activity fails to enhance sexual motivation and blockade of dopamine receptors does not reduce it. In procedures where motivation is estimated via behavior patterns sensitive to enhanced or decreased locomotor activity, results are obviously different (e.g., Graham and Pfaus, 2012). Whether such observations have any relevance for the issue of sexual motivation is another question. It is also important to consider that drugs enhancing dopaminergic neurotransmission increase social motivation (Sharp and Smith, 2022). Thus, data from procedures not including a control for that cannot offer unequivocal evidence for an effect on sexual motivation.

Drugs acting at the adrenergic α_2_ receptor have been shown to alter sexual motivation in male rats. The antagonists yohimbine and RX821002 enhance sexual motivation, whereas agonists like dexmedetomidine and S18616 reduce it (Viitamaa et al., 2006; Chu and Ågmo, 2016). Curiously enough, the reliable motivational effects of drugs acting at the α_2_ receptor have not provoked the slightest interest.

Chronic treatment with a solid dose of paroxetine reduces female rats’ approach to a sexually active male (Kaspersen and Ågmo, 2012). Approach to a social incentive, a castrated male, was unaffected by the drug. Likewise, acute treatment was ineffective. Another serotonin reuptake inhibitor, fluoxetine, has also been shown to lack effect on sexual approach behavior after acute treatment (Marshall et al., 2020). To the contrary, when the dependent variable was nose pokes in an operant procedure in which nose pokes opened the door to a sexually active male, acute fluoxetine reduced the number of nose pokes (Uphouse et al., 2015). Since no control for social motivation was included in this study, the reported effect could be due to impaired social motivation, or simply to reduced locomotor activity, or perhaps to impaired memory of the operant task. The first of these possible explanations is the most likely, because fluoxetine has been shown to reduce the rewarding value of a social stimulus, whereas the reward value of a non-social stimulus (a black-and-white athletic sock of the same size and color as a Long-Evans rat) was unaffected (Sharp and Smith, 2022).

Kisspeptin is known to be important for sexual functions. Pharmacogenetic stimulation of kisspeptin neurons in the posterodorsal, medial amygdala enhances sexual approach behaviors in male mice while leaving social approach unaffected (Adekunbi et al., 2018). We will not make a listing of all transmitters that in some way or another have been reported to affect sexual motivation as estimated through the intensity of approach behavior. Even though sexual approach has attracted far less attention than copulatory behaviors, there is enough data to suggest that neurotransmitters may modify sexual motivation. Since transmitters act on neurons, it may also be proposed that the concept of motivation somehow can be reduced to or materialized in neuronal activity. Although transmitters act on neurons both within and outside the central nervous system, the reports of motivational effects of intracerebral manipulations of kisspeptin activity may mean that central neurons are involved.

Before leaving this section, it is extremely important to note that motivational effects of altered activity of a transmitter in no way indicates that this transmitter normally is involved in the control of motivation. To ascribe a transmitter a role in the physiological (i.e., normal) control of sexual motivation it needs to be shown that the firing activity of the corresponding neurons is altered when the subject is exposed to sexual incentive stimuli, and that the inactivation of these neurons has consequences for sexual motivation. The simple fact that artificially induced alterations in their activity has motivational effects is not sufficient evidence for a role of these neurons in the physiological control of motivation. The frequent error of attributing a physiological function to everything that can modify behavior is a source of much confusion and at the origin of many false hypotheses. Materializing sexual motivation in one or several transmitters at one or several brain sites is a dangerous undertaking, and it should not be endeavored without evidence from multiple sources.

#### Humans

Serotonergic agents, in the form of specific serotonin reuptake inhibitors, have been much used for the treatment of depression, and they have acquired a solid reputation for adverse sexual side effects (Rosen et al., 1999; Atmaca, 2020). However, we have not been able to find a single study in which the effects of serotonergic agents on genital responses to sexual incentives have been evaluated. This precludes any serious discussion of serotonin effects on human sexual motivation.

The only transmitter that has been manipulated in studies where genital responses have been recorded is noradrenaline. The non-selective adrenergic agonist ephedrine, at a dose of 50 mg, enhanced the vaginal response to a pornographic video segment without affecting the response to a neutral video (Meston and Heiman, 1998). However, the selective α_2_ agonist clonidine, at a dose of 0.2 mg, had no effect (Meston et al., 1997). Moreover, the Chu and Ågmo (2016) antagonist yohimbine, at a dose of 6 mg, failed to alter the vaginal response to a pornographic video segment in young women (Meston and Worcel, 2002). Unfortunately, a study employing another non-selective agonist, phentolamine, at the solid dose of 40 mg, failed to alter the vaginal response to a pornographic movie (Rosen et al., 1999). Nevertheless, the fact that ephedrine did enhance the response, hence sexual motivation, suggests that neurons somehow control the behavioral manifestations of this concept. It is most likely that the effects of ephedrine are purely pharmacological, not suggesting any role of the α receptors in the physiological control of sexual motivation. Whether ephedrine acts centrally or peripherally when producing its effect on vaginal responses is unknown.

In men, there are no objective data concerning drug effects on penile responses to sexual incentives although there are hosts of questionnaire studies (e.g., Trinchieri et al., 2021). We conclude by stating that sexual motivation in women can be altered by molecules affecting the nervous system. We do not know how and if sexual motivation in men involves the nervous system at all.

#### Sexual motivation materialized at specific brain sites as revealed by lesion or stimulation

There is an extensive literature concerning the effects of lesions of all kinds on copulatory behavior. At the same time, there is a scarcity of studies of sexual approach behaviors following lesions of the brain. We will now briefly mention these studies.

The first to show that electrolytic lesion of the medial preoptic area in male rats eliminated preferential approach to a sexually receptive female was David Edwards at Emory (Edwards and Einhorn, 1986). Later it was found that preoptic lesion also eliminates male rats’ preferential approach to female odors (Dhungel et al., 2011). Moreover, silencing of the preoptic area with local administration of a sodium channel blocker eliminates the preferential approach to a sexual incentive without modifying approach to a social incentive or altering motor activity (Hurtazo et al., 2008). These examples should be sufficient for concluding that sexual motivation in male rats is materialized within the preoptic area. The fact that Dhungel et al. (2011) used ibotenic acid for making the lesions also shows that neurons and not glia is the site of sexual motivation.

Another brain site that has attracted attention is the bed nucleus of the stria terminalis. Again, David Edwards was the first to show that lesion of this nucleus eliminated preferential approach to a sexually receptive female (Edwards et al., 1996). In male hamsters, electrolytic lesion of the bed nucleus of the stria terminalis reduced the time spent investigating vaginal secretions from sexually receptive females (Powers et al., 1987). Unfortunately, there was no control for approaches to other odors. Perhaps the lesioned hamster would have shown reduced approached to any odor.

More recent data from sexually inexperienced male hamsters show that excitotoxic lesion (400 ng of N-methyl-d-aspartic acid bilaterally) of the posterior part of the bed nucleus of the stria terminalis reduces approach to a sexually receptive female whereas approach to a male is unaffected. In experienced males, the lesion had no effect (Been and Petrulis, 2010). These data could suggest that the central nervous localization of the concept of sexual motivation depends on the subject’s state of virginity. We will return to this delicate proposal below.

There is also preliminary evidence for a role of the bed nucleus of the stria terminalis, principal component, in male mice approach to urine from sexually receptive females. Aromatase expressing neurons within this area increase firing rate when sexually inexperienced mice are exposed to this stimulus, and chemogenetic silencing of these neurons abolishes the males’ preferential approach to female urine (Bayless et al., 2019). We have already mentioned the problems caused by the use of urine as sexual incentive, making the relevance of the Bayless et al. (2019) data for sexual approach behaviors unclear. Furthermore, the exclusive use of sexually inexperienced males was most unfortunate. A sexual encounter in animals lacking sexual experience is a once-in-a-lifetime event, and the overwhelming majority of sexual interactions occurs between experienced animals. Even though humans attach great importance to the loss of virginity, particularly in women, this event is probably less overturning in other animals. Thus, rather than providing data from an unusual situation, it would be more interesting with data relevant for most sexual activity. Nevertheless, the Bayless et al. (2019) study is exemplar in its use of molecular techniques for selectively studying neurons expressing aromatase, even though their data do not provide any clue to the functions of this enzyme in these neurons. Nevertheless, despite these limitations we suggest that neurons in the bed nucleus of the stria terminalis may be one of the sites where the concept of sexual motivation is materialized in male rats, hamsters and mice.

Electrolytic lesion of the medial amygdala in female hamsters eliminates the preferential approach to odors from an intact male. In fact, the females did not approach the male odors more than the odor from another female (Petrulis and Johnston, 1999). The lesion did not reduce approach to the female odor. The specific reduction in the approach to a sexually relevant stimulus suggests that sexual motivation is eliminated when the medial amygdala is destroyed. The problems associated with the unfortunate habit of replacing living animals with odors collected from such animals has already been discussed several times.

Excitotoxic lesion of the bed nucleus of the stria terminalis in female hamsters had no effect on approach to odors from conspecifics. Male odor was far more approached than female odor in lesioned as well as control hamsters (Martinez and Petrulis, 2011). The conclusion to be drawn from this short summary of some observations on the effects of brain lesions on sexual motivation is that while specific brain sites may be of importance, that importance is usually limited to specific circumstances, for example lack of sexual experience, or the sexual incentive stimulus being a living conspecific or the odor of excreta from a conspecific.

#### Hormonal, neurochemical, and structural conclusion

Several pages ago we showed that gonadal hormones may alter the level of sexual motivation. We also saw that pharmacological manipulations of some neurotransmitters may have some modest effect on sexual motivation. Both these effects are likely mediated by neurons, although direct evidence for that is lacking in humans. Likewise, many of the lesion effects can be attributed to death of neurons rather than glia. Thus, converging evidence show that the concepts of sexual motivation and central motive state have a neural basis, most probably located to the brain. Furthermore, the level of sexual motivation can be altered by neurobiological manipulations as well as by removal of the gonads, and probably many other things.

## To what use can we put the materialized sexual motivation?

An indispensable requisite for any concept to be acceptable in empirical science is that it can be quantified. We have shown that sexual motivation, or the activity level of the central motive state, can be quantified as the intensity of sexual approach behavior in rodents and as the magnitude of the genital response in humans. Thus, the quantification criterion is satisfied. In addition to quantification, abstract concepts must be anchored to the physical world in one way or another. We have seen that sexual motivation can be altered by molecules, elements of physical reality, and the action of some of these molecules can be localized to neurons in the brain. The basic element of neural function is the firing of action potentials. Thus, the concept of motivation can, in principle, be reduced to action potentials. Some molecules may have actions outside the brain, and some even in non-nervous tissue, but this does not reduce the connection between concept and physical reality. Nevertheless, physical events other than action potentials may be part of sexual motivation.

Having established that motivation and central motive state have a physical, and perhaps partly neural, substrate leads to another question: So what? In which way does the material existence of these concepts make them more useful as explanatory tools than their status as intervening variables? This question is crucial for many of the concepts used in the behavioral sciences, like habituation, sensitization, expectations, executive functions, reward, and so on.

The obvious answer to the question posed in the preceding paragraph is that the physical existence of a concept makes it amenable to manipulation by elements in the physical world. If we accept that sexual motivation is anchored in neurons in the brain, then manipulations of these neurons could alter motivation. For example, a drug could enhance it or reduce it, modified gene expression could do the same, as could optogenetic inhibition or stimulation of neuronal firing. In the earlier sections of this review, we have mentioned that all these means to alter nervous activity have been employed in studies of non-human animals, often with varying and contradictory results. However, despite the unclear picture of the control of sexual motivation coming out from these studies, it must be recognized that the simple fact of making the subject accessible to scientific inquiry is a major contribution of having materialized it.

The material nature of sexual motivation has also led to efforts to manipulate human sexual motivation. For centuries, men have dreamed of having an aphrodisiac to drop in the glass of an attractive woman, making her sexual motivation so intense that she would agree to sexual intercourse. Likewise, ailing men were hoping to boost their sexual motivation with a powerful concoction. Aphrodisiacs were probably never regarded as socially acceptable, but after the inclusion of the diagnosis hypoactive sexual desire in the revision of the 3^rd^ edition of the Diagnostic and Statistical Manual of the American Psychiatric Association (1987) it became legitimate to search for a drug with aphrodisiac action. Obviously, the desired drug was not intended to work as an aphrodisiac, but in a magic way only enhance sexual motivation in women having received the appropriate diagnosis. Men were excluded as targets for drugs enhancing sexual motivation. Nevertheless, the simple fact that the large pharmaceutical companies and many respectable scientists engaged themselves in the search for a molecule enhancing sexual motivation in women shows that they all believed in a material basis of the concept of motivation. The meager results of the search for drugs enhancing sexual motivation in women, mentioned already in the introduction, is another story and does not necessarily suggest that sexual motivation is immaterial, beyond the reach of molecules.

## Causes of the slow progress in understanding the material bases of sexual motivation

The many inconsistencies in the data emanating from research on sexual motivation in rodents can be attributed to poor operational definitions. Even recent, sophisticated studies of the neural circuitry of sexual behaviors employ a medley of unvalidated tests for motivation, not preventing the authors from making far-reaching assertions (e.g., Bayless et al., 2023). Parameters of copulatory behavior are often imputed with motivational meaning, even when much evidence show that they depend on non-motivational factors (reviewed in Le Moëne and Ågmo, 2019). Likewise, the tests of approach behavior based on demanding motor tasks produce confounds between effects of motor systems and effects on sexual motivation, as pointed out before. Moreover, scientists are usually very careful when interpreting the results of the statistical evaluation of their data while forgetting that the crucial element is the validity of the verbal hypothesis they are testing. If the hypothesis being tested is “drug D enhances sexual motivation in female rats” and the data used for the tests are the number of paracopulatory behaviors displayed per unit time, we are in serious problems. A statistically significant effect would be interpreted as a significant increase in sexual motivation, whereas it was only found that the frequency of paracopulatory behavior was increased. The relationship between these behaviors and sexual motivation is entirely unknown, yet some scientists allow themselves the metamorphosis described above. The problems with testing verbal hypothesis transformed into numbers with unknown meaning has led to what is known as the generalizability crisis in psychology (Yarkoni, 2022). These and many other factors (see Hernández-Arteaga and Ågmo, 2023) delay progress in our understanding of sexual motivation in non-human animals.

In humans, the anchoring of sexual motivation to the brain is less convincing than in other animals. Very little usable data for distinguishing peripheral and central actions of drugs and hormones are available. Despite this, it seems to be a general belief that human sexual motivation is determined by events in the brain. Unfortunately, efforts to modify brain function have so far left sexual motivation unaffected.

In recent decades, there has been a growing interest in studying brain activity using functional magnetic resonance imaging (fMRI). The lack of standardized methodologies and paradigms in sexual research makes it difficult to reach a consensus between disparate reports (Mills-Finnerty et al., 2022). Nevertheless, some reports have shown correlations between penile erection and BOLD response in areas such as the supramarginal gyrus as well as in visual areas including the temporal and occipital cortices. BOLD activity in these areas has been associated with the visual stimuli presented during those studies, as well as with the arousing features of the materials used to induce sexual responses. BOLD responses in the cingulate gyrus and insula are also correlated with genital arousal in both sexes. These areas have been attributed to interoceptive awareness of bodily states, including genital awareness.

Additional areas, such as the anterior and posterior cingulate cortex, show enhanced BOLD signal during genital arousal in women (Seok et al., 2016; Parada et al., 2018). However, the exact role of these areas in sexual arousal is unclear. As previously noted, the current state of fMRI studies on genital arousal are far from conclusive and are open to broad interpretations. In fact, coincidences between hemodynamic responses in some brain areas and genital responses cannot be used to establish any cause-effect relationship. It is quite possible that altered BOLD signals are epiphenomena without any functional significance, albeit useful for building sophisticated models and generating extravagant hypotheses. Because of these reasons we do not consider brain imaging data to be relevant for the present review.

The main problem in human studies, as well as in many studies in other animals, seems to be poor operationalization of sexual motivation. The many drug trials performed have all employed questionnaires for inferring alterations in motivation, but not a single study has evaluated the genital response to sexual incentives. Under these circumstances, there is not much reason to believe that any efficient drug ever will be found. Most unfortunately, few or none of the many concepts used in the questionnaires pretending to evaluate sexual motivation have been connected to the material world, and their possible relationship to sexual motivation has usually no foundation except the researcher’s intuition. Evaluating drug actions employing non-material (esoteric?) concepts is a hazardous enterprise, destined to failure.

## General conclusion

To conclude this review, we propose that research on sexual motivation always should be based on objective and quantitative measurements of variables shown to depend on the intensity of sexual motivation, regardless of whether the experimental subject is human or non-human. By following this proposal, it should be possible to unravel the material bases for sexual motivation. After having done so, it may become pertinent to search for reliable ways to alter that motivation in the desirable direction. At present, however, such a search is premature.

## Author contributions

EV-A: Writing – original draft. AÅ: Writing – original draft.
